# CT Morphological Features Integrated With Whole-Lesion Histogram Parameters to Predict Lung Metastasis for Colorectal Cancer Patients With Pulmonary Nodules

**DOI:** 10.3389/fonc.2019.01241

**Published:** 2019-11-19

**Authors:** TingDan Hu, ShengPing Wang, Xiangyu E, Ye Yuan, Lv Huang, JiaZhou Wang, DeBing Shi, Yuan Li, WeiJun Peng, Tong Tong

**Affiliations:** ^1^Department of Radiology, Fudan University Shanghai Cancer Center, Shanghai, China; ^2^Department of Oncology, Shanghai Medical College, Fudan University, Shanghai, China; ^3^Department of Radiotherapy, Fudan University Shanghai Cancer Center, Fudan University, Shanghai, China; ^4^Department of Colorectal Surgery, Fudan University Shanghai Cancer Center, Fudan University, Shanghai, China; ^5^Department of Pathology, Fudan University Shanghai Cancer Center, Fudan University, Shanghai, China

**Keywords:** colorectal cancer, pulmonary metastases, histogram, morphological, morphological features, nomogram

## Abstract

**Purpose:** To retrospectively identify the relationships between both CT morphological features and histogram parameters with pulmonary metastasis in patients with colorectal cancer (CRC) and compare the efficacy of single-slice and whole-lesion histogram analysis.

**Methods:** Our study enrolled 196 CRC patients with pulmonary nodules (136 in the training dataset and 60 in the validation dataset). Twenty morphological features of contrast-enhanced chest CT were evaluated. The regions of interests were delineated in single-slice and whole-tumor lesions, and 22 histogram parameters were extracted. Stepwise logistic regression analyses were applied to choose the independent factors of lung metastasis in the morphological features model, the single-slice histogram model and whole-lesion histogram model. The areas under the curve (AUC) was applied to quantify the predictive accuracy of each model. Finally, we built a morphological-histogram nomogram for pulmonary metastasis prediction.

**Results:** The whole-lesion histogram analysis (AUC of 0.888 and 0.865 in the training and validation datasets, respectively) outperformed the single-slice histogram analysis (AUC of 0.872 and 0.819 in the training and validation datasets, respectively) and the CT morphological features model (AUC of 0.869 and 0.845 in the training and validation datasets, respectively). The morphological-histogram model, developed with significant morphological features and whole-lesion histogram parameters, achieved favorable discrimination in both the training dataset (AUC = 0.919) and validation dataset (AUC = 0.895), and good calibration.

**Conclusions:** CT morphological features in combination with whole-lesion histogram parameters can be used to prognosticate pulmonary metastasis for patients with colorectal cancer.

## Introduction

Colorectal cancer (CRC) is the third common cause of morbidity and mortality worldwide (1, 2). Pulmonary is the most common extra-abdominal site of metastasis for those with CRC, with 5–10% of CRC patients developing pulmonary metastasis (PM) (3, 4). The 5-year survival rates after initial colorectal surgery in patients with and without resection for pulmonary metastasis are 68 and 13%, respectively (3). The strong survival benefits of pulmonary metastasectomy make this treatment the generally accepted treatment for patients to achieve long-term survival when there is a definite and clear diagnosis (5, 6). Furthermore, if pulmonary metastasis is diagnosed early and resected aggressively, the survival rate is further improved (7).

However, with chest CT applied as part of preoperative routine examination, an increasing number of CRC patients are being diagnosed with indeterminate pulmonary nodules (IPNs) of unknown nature (8). The reported incidence of IPNs in CRC patients is 25–45.5% (8–10). Further diagnostic tests can also be problematic as nodules <10 mm in diameter may fall below the threshold of detection for positron emission tomography (PET) (11), and fine-needle aspiration cytology may not be feasible for thoracoscopic localization (12). Therefore, in CRC patients with IPNs, the accurate diagnosis of metastatic disease at an early and surgically treatable stage remains a challenge.

Though early-stage metastatic nodules and benign lesions have similar appearance in images, the importance of morphology should not be underestimated (13). CT imaging allows detailed observation of the morphological features of nodules and lesions, such as their internal density, shape, margin, and other typical characteristics. In recent years, texture analysis has emerged as a valuable methodology for facilitating diagnosis through the deep mining of information from medical images (14, 15). It has achieved great utility in evaluating many kinds of pulmonary diseases, including pulmonary embolisms (16), interstitial lung disease (17), and pulmonary nodules (18, 19). By extracting features of subtle pixel distributions and spatial variations of the gray levels of lesions that are imperceptible to the naked eye, texture analysis provides a complementary method for evaluating subjective and megascopic morphological features.

To date, studies concentrating on the morphological and textural features of IPNs 5–20 mm in diameter on contrast-enhanced CT in CRC patients remain limited. This study sought to determine the morphological characteristics and histogram parameters derived from texture analysis for CRC patients with IPNs and to construct a risk model with a combination of independent predictors to facilitate the accurate diagnosis of pulmonary metastasis.

## Materials and Methods

### Patients

This retrospective analysis had obtained the ethical approval, and the informed consent requirement was waived. Our study enrolled 196 consecutive colorectal cancer patients (88F/108M; age range, 32–80 years; mean age, 58.49 ± 10.80 years) with lung nodules admitted in our institution between January 2010 and December 2017. The inclusion criteria were as follows: (i) colorectal cancer was histopathologically confirmed; (ii) at least one lung nodule measuring 5–20 mm detected by contrast-enhanced chest CT examination; (iii) available pathology reports with diagnosis of pulmonary metastasis or primary lung cancer for the malignant nodules and at least 2 years follow*-*up for the benign nodules; and (iv) complete medical history. The exclusion protocol were as follows: (i) with pretreatment 6 months before initial CT examination (including chemotherapy or pneumonectomy); (ii) obsolete nodules detected 6 months before colorectal cancer was detected; (iii) obvious benign nodules with typical imaging characteristics (such as cysts, tuberculosis, or inflammatory nodules); and (iv) adjuvant therapy (including radiation therapy or chemotherapy) applied for no-progress lesions in the process of follow*-*up. When there are multiple nodules, we choose the largest nodule for morphological and radiomics analysis. Of the 196 people included in the study, 194 of them have been published in our previous research (20).

**Figure 1:**
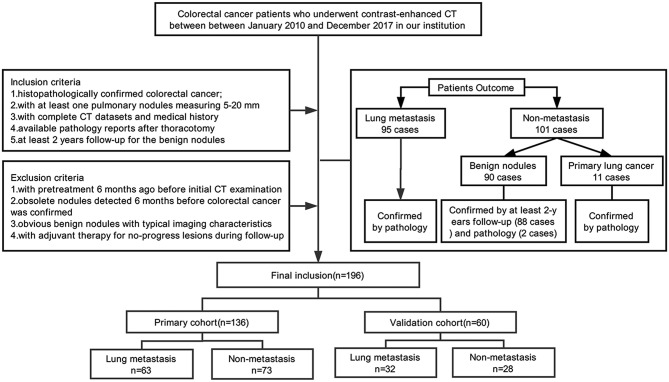
Flow chat of patients' recruitment pathway.

Nodules were divided into two groups: (i) a pathologically confirmed lung metastasis group (95 PMs; 42F/53M; mean age, 57.46 ± 10.58 years), and (ii) a non-metastasis (NM) group (101 NMs; 46F/55M; mean age, 59.47 ± 10.91 years), including benign nodules (90 cases) with at least 2 years follow*-*up (88 cases) and pathology confirmation (2 cases) or primary lung cancer confirmed by pathology (11 cases). We used a computer algorithm to randomly divide the patients into a training dataset and a validation dataset at the ratio of 7:3. Figure 1 shows the process of patients' recruitment.

### CT Scanning Protocol

Chest CT examinations were performed at our institution with the Sensation 64 scanner (Siemens Healthcare) or the Somatom Definition AS scanner (Siemens Healthcare). The Contrast-enhanced CT scan parameters were as follows: contrast medium, inhexol; tube voltage, 120 kVp; tube current, 250–350 mA; slice thickness, 1.5 mm; slice interval, 1.5 mm; matrix, 512 × 512; field of view (FOV, 35–50 cm; pitch, 1.078; reconstruction algorithm, standard. The arterial phase of the target nodule which was pathologically confirmed or under follow-up was selected for reconstruction.

### CT Image Interpretation

The interpretations of CT features are listed in Supplementary Table 1. The CT morphological features were independently evaluated by two operators (SW and TH, with 20 and 3 years of experience in chest CT, respectively). In cases of disagreement, a third radiologist (TT, with 20 years of experience in CT imaging) was consulted, and the majority value was used. Mean values were calculated for continuous variables. The CT images were read with both mediastinal and lung window settings. All of the operators were blinded to the clinical and histologic findings.

### Histogram Analysis

Reconstructed images were transferred to the MIM software (v6.6.3; MIM Software Inc.) for histogram analysis. For each patient, regions of interest (ROIs) were first semi-automatically contoured in the largest-cross sectional area of the tumor outline and then manually delineated by an operator and verified by an expert radiologist. Each ROI was propagated to include the entire tumor volume in each consecutive slice using the same contouring method. In the process of delineation, we excluded the border of the lesion and any other irrelevant tissues or regions, such as pleura, normal tissue, air, peripheral vessels, and surrounding organs. Supplementary Figure 1 shows an example of ROI delineation.

The histogram parameters were automatically measured by the software using a volumetric approach on the ROI of the nodule. Single-slice and whole-lesion histogram parameters were extracted and analyzed. From each segmented tumor, we extracted 11 single-slice histogram parameters and 11 whole-lesion histogram features. More information about the methodology used to extract histogram features can be found in Supplementary Material.

### Statistical Analysis

R software (version 3.3) was applied for statistical analysis. To measure the agreement of CT morphological features between two readers, intraclass correlation coefficients (ICCs) were calculated (poor: 0.00–0.20; fair: 0.21–0.40; moderate: 0.41–0.60; good: 0.61–0.80; excellent: 0.81–1.00). To compare the proportional differences between the training dataset and the validation dataset, chi-square tests were applied for the categorical variables, and two-sample *t*-tests were used for the continuous variables. To compare the differences between the PM and NM group, chi-square and two-sample *t*-tests were applied as appropriate for both the training and validation datasets. Two-sided *p* < 0.05 was considered significant.

### Model Selection

The significant factors were introduced into the stepwise logistic regression to select the independent features for the CT morphological model, the single-slice histogram model and the whole-lesion histogram model. The Akaike information criterion (AIC) was employed as the stopping rule. The validation dataset was used to test the diagnostic performance of the models by applying the multivariable regression formula derived from the training dataset to the patients of the validation dataset, and the probability of metastasis was calculated for each. The area under the receiver characteristic curve (AUC) was calculated to quantify the predictive accuracy of the three models in the training and validation datasets. We also calculated the accuracy, sensitivity, specificity, positive predictive value, and negative predictive value for each model.

We compared the relative strengths of the single-slice and whole-lesion histogram models and then used the more efficient model in combination with the morphological features to construct the morphological-histogram model. A morphological-histogram nomogram was then constructed for clinical application. A receiver operating characteristic (ROC) curve was used to describe the discrimination abilities of the nomogram. An AUC above 0.75 is considered as good (21). Nomogram performance was graphically demonstrated by calibration plots in both the training and validation datasets. Finally, decision curve analysis (DCA) was applied to assess the clinical usefulness of the nomogram.

## Results

### Patient Characteristics

The patients characteristics and statistically significant CT morphological features are shown in Table 1 (Supplementary Table 3 contains complete morphological features comparison), and the histogram parameters are presented in Table 2. There were no significant differences between the training and validation datasets except in pleural attachment (Supplementary Table 2). The agreement between the two operators was excellent for most characteristics and good for several features (Supplementary Table 4).

**Table 1:** Comparison of morphological features of lung metastasis (PM) and non- metastasis (NM) in the training and validation datasets.

	**Training dataset**			**Validation dataset**		
**Characteristics**	**NM**	**PM**	***p***	**NM**	**PM**	***p***
Age	59.82 ± 10.530	58.17 ± 11.360	0.382	58.54 ± 12.188	56.06 ± 9.055	0.372
Gender			0.676			0.316
1	42 (57.5%)	34 (54.0%)		13 (46.4%)	19 (59.4%)	
2	31 (42.5%)	29 (46.0%)		15 (53.6%)	13 (40.6%)	
Lobe location			0.025^*^			0.507
1	12 (16.4%)	19 (30.2%)		7 (25.0%)	6 (18.8%)	
2	12 (16.4%)	10 (15.9%)		4 (14.3%)	10 (31.3%)	
3	23 (31.5%)	9 (14.3%)		8 (28.6%)	5 (15.6%)	
4	12 (16.4%)	5 (7.9%)		1 (3.6%)	1 (3.1%)	
5	14 (19.2%)	20 (31.7%)		8 (28.6%)	10 (31.3%)	
Size category			<0.001^*^	0.00%	0.00%	0.095
1	56 (76.7%)	22 (34.9%)		20 (71.4%)	14 (43.8%)	
2	10 (13.7%)	24 (38.1%)		4 (14.3%)	10 (31.3%)	
3	7 (9.6%)	17 (27.0%)		4 (14.3%)	8 (25.0%)	
Long-axis diameter	8.988 ± 3.493	12.662 ± 4.200	<0.001^*^	9.221 ± 4.422	11.819 ± 4.180	0.023^*^
Short-axis diameter	5.825 ± 2.144	9.611 ± 9.253	0.001^*^	5.682 ± 2.031	8.184 ± 3.071	<0.001^*^
Density			<0.001^*^			0.002^*^
1	12 (16.4%)	0 (0.0%)		6 (21.4%)	0 (0.0%)	
2	15 (20.5%)	2 (3.2%)		6 (21.4%)	2 (6.3%)	
3	46 (63.0%)	61 (96.8%)		16 (57.1%)	30 (93.8%)	
Contour			0043^*^			0.011^*^
1	2 (2.7%)	10 (15.9%)		1 (3.6%)	1 (3.1%)	
2	21 (28.8%)	20 (31.7%)		2 (7.1%)	8 (25.0%)	
3	29 (39.7%)	19 (30.2%)		11 (39.3%)	19 (59.4%)	
4	21 (28.8%)	14 (22.2%)		14 (50.0%)	4 (12.5%)	
Border			<0.001^*^			<0.001^*^
1	28 (38.4%)	2 (3.2%)		13 (46.4%)	1 (3.1%)	
2	24 (32.9%)	43 (68.3%)		10 (35.7%)	20 (62.5%)	
3	21 (28.8%)	18 (28.6%)		5 (17.9%)	11 (34.4%)	
Air bronchogram			0.032^*^			0.178
0	72 (98.6%)	57 (90.5%)		28 (100.0%)	30 (93.8%)	
1	1 (1.4%)	6 (9.5%)		0 (0.0%)	2 (6.3%)	
Lymphadenopathy			0.032^*^			0.369
0	72 (98.6%)	57 (90.5%)		27 (96.4%)	29 (90.6%)	
1	1 (1.4%)	6 (9.5%)		1 (3.6%)	3 (9.4%)	

*Chi-square tests were used to compare the differences in categorical variables while a two-sample t-test was used to compare the differences in continuous variables*.

*NM, non-metastasis group; PM, lung metastasis group*.

* *p < 0.05*.

**Table 2:** Comparison of single-slice and whole-lesion histogram parameters of PM and NM in the training and validation datasets.

**Parameters**	**Training dataset**			**Validation dataset**		
	**NM**	**PM**	**p**	**NM**	**PM**	***p***
S-ASD	−2.976 ± 15.859	−2.902 ± 8.077	0.973	−2.312 ± 9.593	0.490 ± 6.293	0.181
S-STD	161.602 ± 101.977	135.420 ± 60.213	0.076	129.270 ± 68.500	130.685 ± 59.258	0.932
S-Average ratio	0.428 ± 0.685	1.345 ± 1.414	<0.001^*^	0.523 ± 0.888	0.993 ± 0.887	0.045^*^
S-Mean	−58.878 ± 340.285	−15.669 ± 330.362	0.456	−73.787 ± 164.636	−37.878 ± 88.316	0.289
S-Skewness	0.088 ± 1.089	−0.854 ± 0.816	<0.001^*^	0.231 ± 1.035	−0.871 ± 0.995	<0.001^*^
S-Kurtosis	0.987 ± 4.616	1.163 ± 3.397	0.802	1.364 ± 3.860	1.727 ± 5.520	0.772
S-Area	0.775 ± 0.357	1.236 ± 0.416	<0.001^*^	0.795 ± 0.419	1.128 ± 0.373	0.002^*^
S-Volume	0.428 ± 0.685	1.345 ± 1.414	<0.001^*^	0.523 ± 0.888	0.993 ± 0.887	0.045^*^
S-Median	−50.810 ± 344.826	−73.250 ± 146.112	<0.001^*^	−299.820 ± 296.139	−29.190 ± 114.492	<0.001^*^
S-Maximum	190.590 ± 450.767	156.170 ± 176.689	0.57	123.320 ± 600.592	197.130 ± 112.848	0.498
S-Minimum	−58.970 ± 233.980	−69.970 ± 252.732	0.035^*^	−623.040 ± 210.656	−532.810 ± 289.115	0.178
W-ASD	364.124 ± 909.629	218.649 ± 176.266	0.214	211.019 ± 314.358	204.203 ± 179.826	0.917
W-STD	161.978 ± 101.572	135.300 ± 60.134	0.07	128.909 ± 68.194	130.383 ± 59.421	0.929
W-Average ratio	0.960 ± 0.466	1.610 ± 0.522	<0.001^*^	0.960 ± 0.404	1.485 ± 0.450	<0.001^*^
W-Mean	−39.095 ± 326.163	−99.504 ± 129.099	0.002^*^	−292.437 ± 290.740	−56.853 ± 106.652	<0.001^*^
W-Skewness	0.064 ± 1.016	−0.851 ± 0.824	<0.001^*^	0.245 ± 1.041	−0.859 ± 1.008	<0.001^*^
W-Kurtosis	3.723 ± 4.279	4.185 ± 3.455	0.494	4.373 ± 3.957	4.713 ± 5.622	0.791
W-Area	86.586 ± 145.308	206.406 ± 221.798	<0.001^*^	93.797 ± 112.534	167.841 ± 189.459	0.076
W-Volume	134.501 ± 340.853	394.708 ± 510.214	0.001^*^	122.925 ± 184.577	301.584 ± 380.789	0.028^*^
W-Median	−42.164 ± 337.581	−73.365 ± 146.188	<0.001^*^	−299.839 ± 295.991	−29.359 ± 114.730	<0.001^*^
W-Maximum	188.810 ± 450.066	156.140 ± 176.460	0.589	138.640 ± 586.255	195.940 ± 110.947	0.59
W-Minimum	−18.330 ± 250.784	−57.700 ± 263.027	0.172	−613.680 ± 225.487	−505.530 ± 311.857	0.134

*ASD, Average standard deviation ratio; STD, Standard deviation*.

*S-, single-slice histogram parameters; W-, whole-lesion histogram parameters. A two-sample t-test was used to compare the differences of those parameters*.

* *p < 0.05*.

### Significant Morphological Features and Histogram Parameters

Regarding the CT morphological features, the chi-square tests and *t*-tests revealed that nine CT features were associated with lung metastasis, including lobe location (*p* = 0.025), size category (*p* < 0.001), long-axis diameter (*p* < 0.001), short-axis diameter (*p* = 0.001), density (*p* < 0.001), contour (*p* = 0.043), border (*p* < 0.001), air bronchogram (*p* = 0.032), and lymphadenopathy (*p* = 0.032). After stepwise logistic analysis, long-axis diameter (OR = 1.360, 95%CI: 1.198–1.544, *P* < 0.001), density (OR = 11.166, 95%CI: 2.721–45.815, *P* < 0.001) and contour (OR = 0.317, 95%CI: 0.177–0.569, *P* = 0.001) remained independent predictors in the CT morphological model, as shown in Table 3.

**Table 3:** Comparison of the models by multivariate logistic regression analysis.

	**OR (95%CI)**	**P**	**AIC**
CT morphological features			127.34
Long-axis diameter	1.360 (1.198–1.544)	<0.001^*^	
Density	11.166 (2.721–45.815)	<0.001^*^	
Contour	0.317 (0.177–0.569)	0.001^*^	
Single-slice histogram			130.90
S-Average ratio	0.268 (0.111–0.642)	0.003^*^	
S-Area	559.372 (42.344–7389.333)	<0.001^*^	
S-Median	1.004 (1.002–1.005)	<0.001^*^	
Whole-lesion histogram			130.25
W-Average ratio	12.764 (4.653–35.018)	<0.001^*^	
W-Mean	0.977 (0.961–0.994)	0.004^*^	
W-Median	1.024 (1.008–1.041)	0.009^*^	
Morphological-histogram			121.74
Density	5.434 (1.161–25.440)	0.032^*^	
Contour	0.495 (0.286–0.858)	0.012^*^	
W-Average ratio	9.727 (3.538–26.740)	<0.001^*^	
W-Mean	0.977 (0.959–0.995)	0.009^*^	
W-Median	1.023 (1.006–1.042)	0.013^*^	

*OR, odds ratio; CI, confidence interval; AIC, Akaike information criterion*.

* *p < 0.05*.

Regarding the single-slice histogram parameters (S- means the parameters from the single-slice histogram analysis and W- from the whole-slice histogram), *t*-tests revealed that the S-average ratio (*p* < 0.001), S-skewness (*p* < 0.001), S-area (*p* < 0.001), S-volume (*p* < 0.001), S-median (*p* < 0.001), and S-minimum (*p* = 0.035) were significant variables related to PM. After stepwise logistic analysis, the S-average ratio (OR = 0.268, 95%CI: 0.111–0.642, *P* = 0.003), S-area (OR = 559.372, 95%CI: 42.344–7389.333, *P* < 0.001), and S-median (OR = 1.004, 95%CI: 1.002–1.005, *P* < 0.001) were selected as independent predictors for the single-slice histogram model.

**Figure 2:**
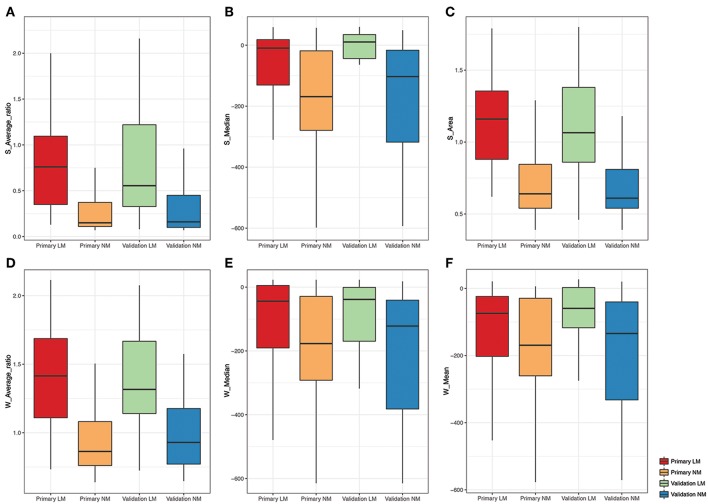
Boxplot of the selected histogram parameters in the LM and NM group. **(A-C)** Boxplot of the S-Average ratio, S-Median, S-Area from the single-slice histogram in the LM and NM group, respectively. **(D-F)** Boxplot of the W-Average ratio, W-Median, W-Mean from the whole-lesion histogram in the LM and NM group, respectively.

Regarding the whole-lesion histogram parameters, *t*-tests revealed that the W-average ratio (*p* < 0.001), W-mean (*p* = 0.002), W-skewness (*p* < 0.001), W-area (*p* < 0.001), S-volume (*p* = 0.001), W-median (*p* < 0.001), and S-minimum (*p* = 0.035) were significant parameters associated with PM. After stepwise logistic analysis, the W-average ratio (OR = 12.764, 95%CI: 4.653–35.018, *P* = 0.003), W-mean (OR = 0.977, 95%CI: 0.961–0.994, *P* = 0.004), and S-median (OR = 1.024, 95%CI: 1.008–1.041, *P* = 0.009) were selected as independent predictors for the whole-lesion histogram model. Figure 2 shows the distributions of the significant histogram parameters in the training and validation datasets.

### Comparison of Single-Slice and Whole-Lesion Histogram Analyses

**Figure 3:**
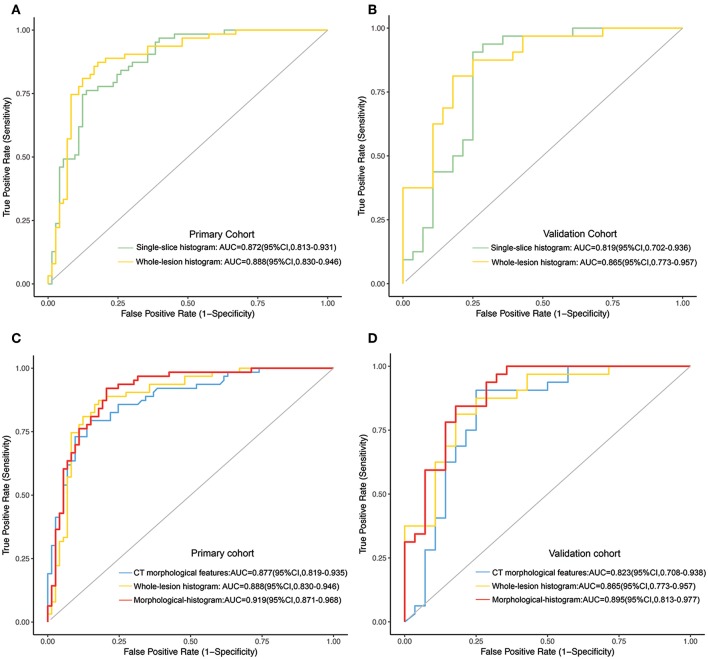
**(A,B)** The ROC curves of the single-slice histogram model and whole-lesion histogram model in the training **(A)** and validation sets **(B)**, respectively. **(C,D)** The ROC curves of the CT morphological model, the whole-lesion histogram model and the integrated morphological-histogram model in the training **(C)** and validation cohorts **(D)**, respectively.

The whole-lesion histogram model (AIC = 130.25) had lower AIC value than the single-slice model (AIC = 130.9) and achieved better discrimination. It yielded an AUC of 0.888 for the training dataset and of 0.865 for the validation dataset, exceeding the AUC values of the single-slice model (AUC = 0.872 for the training dataset and AUC = 0.819 for the validation dataset). The ROC curves of the two models are presented in Figure 3.

### Development and Validation of the Morphological-Histogram Nomogram

We subjected the CT morphological features and the whole-lesion histogram parameters to stepwise logistic regression analysis. Density (OR = 5.434, 95%CI: 1.161–25.440, *P* = 0.032), contour (OR = 0.495, 95%CI: 0.286–0.858, *P* = 0.012), the *W-*average ratio (OR = 9.727, 95%CI: 3.538–26.740, *P* < 0.001), W-mean (OR = 0.977, 95%CI: 0.959–0.995, *P* = 0.009), and W-median (OR = 1.023, 95%CI: 1.006–1.042, *P* = 0.013) were identified as independent risk factors in the model. The integrated model also achieved the best performance among the models, with an AUC of 0.919 (95%CI: 0.871–0.968, accuracy: 88.2%, sensitivity: 84.9%, specificity: 92.1%, PPV: 92.5%, NPV: 84.1%) for the training dataset and of 0.895 (95%CI: 0.813–0.977, accuracy: 81.7%, sensitivity: 78.5%, specificity: 84.4%, PPV: 81.5%, NPV: 81.8%) for the validation dataset (Table 4). The ROC curves of the models for both the training and validation datasets are presented in Figure 3.

**Table 4:** Accuracy and predictive value between those models.

**Training dataset**	**AUC**	**95%CI**	**Sensitivity**	**Specificity**	**Accuracy**	**PPV**	**NPV**
CT morphological features	0.877	0.819–0.935	83.6% (61/73)	79.4% (50/63)	81.6% (111/136)	82.4% (61/74)	80.7% (50/62)
Single-slice histogram	0.872	0.813–0.931	86.3% (63/73)	76.2% (48/63)	81.6% (111/136)	80.8% (63/78)	82.8% (48/58)
Whole-lesion histogram	0.888	0.830–0.946	82.2% (60/73)	87.3% (55/63)	84.6% (115/136)	88.2% (60/68)	80.9% (55/68)
Morphological-histogram	0.919	0.871–0.968	84.9% (62/73)	92.1% (58/63)	88.2% (120/136)	92.5% (62/67)	84.1% (58/69)
**Validation dataset**	**AUC**	**95%CI**	**Sensitivity**	**Specificity**	**Accuracy**	**PPV**	**NPV**
CT morphological features	0.823	0.708–0.938	78.5% (22/28)	75% (24/32)	76.7% (46/60)	73.3% (22/30)	80% (24/30)
Single-slice histogram	0.819	0.702–0.936	75% (21/28)	71.9% (23/32)	73.3% (44/60)	70% (21/30)	76.7% (23/30)
Whole-lesion histogram	0.865	0.773–0.957	75% (21/28)	84.4% (27/32)	80% (48/60)	80.8% (21/26)	79.4% (27/34)
Morphological-histogram	0.895	0.813–0.977	78.5% (22/28)	84.4% (27/32)	81.7% (49/60)	81.5%(22/27)	81.8%(27/33)

*CI, confidence interval; AUC, area under the curve; PPV, positive predictive value; NPV, negative predictive value*.

*p < 0.05*.

**Figure 4:**
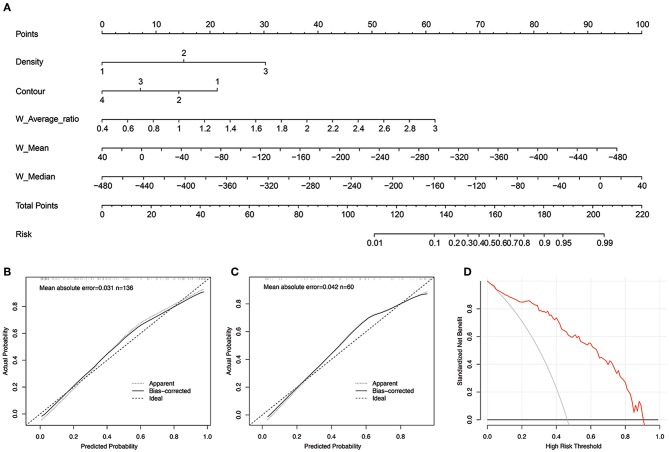
**(A)** The developed morphological-histogram nomogram for predicting the probability of pulmonary metastases. By summing the scores of each point and locating on the total score scale, the estimated probability of pulmonary metastases could be determined. **(B,C)** The Calibration curves for predicting pulmonary metastases in the training and validation cohort. The y axis represents the actual rate of LM. The x axis represents the predicted probability of LM. The ideal line represents a perfect prediction by an ideal model. The apparent line represents the performance of the nomogram model, of which a closer fit to the ideal line represents a better prediction. **(D)** The decision curves analysis for the morphological-histogram nomogram. The red line represents the net benefit of morphological-histogram model. Across the various threshold probabilities, the morphological-histogram curve showed great net benefit.

The morphological-histogram nomogram was successfully constructed, with good discrimination, based on the morphological-histogram model (Figure 4A). The calibration plots also presented good accordance between the nomogram prediction and actual outcome for PM and NM in both the training and validation datasets (Figures 4B,C). The decision curve analysis demonstrated that given a threshold probability ranging from 0 to 100%, the morphological-histogram model was superior to the treat-all and treat-none schemes in predicting lung metastasis (Figure 4D).

## Discussion

In the present study, we investigated the imaging characteristics of IPNs 5–20 mm in diameter on initial CT in CRC patients and compared the predictive accuracy of whole-lesion and single-slice histogram parameters. We then constructed a morphological-histogram nomogram using a combination of morphological features and whole-lesion histogram parameters for IPNs. This nomogram may be clinically useful for discriminating CRC patients who might benefit from early and curable metastasectomy for metastatic lesions or an appropriate surveillance program.

CT offers direct visualization of lesions and potentially allows a detailed characterization of the morphologic extent of lesions. The careful evaluation of morphologic features is an essential step in pulmonary nodules assessment (13). Although several studies (22–24) have sought to identify significant image features for metastatic nodules, there is no consensus regarding the definition of IPNs, which led to slight differences between our results and previously published ones. In our study, we found that significant morphological features associated with pulmonary metastasis were long-axis diameter, density, and contour.

As reported by many other studies, nodule diameter is a reliable indicator of malignant potential (22, 23, 25). We found that solid nodules are more likely to be metastatic lesions. As more than 95% of nodules that originate from colorectal cancer are adenocarcinomas (4), metastatic lesions tend to appear as solid pulmonary nodules (SPN) in CT scans, whereas benign lesions, such as inflammation lesions, or organizing pneumonia/fibrosis consistently present as patchy consolidations or mixed-density regions surrounded by ground-glass opacity (GGO) owing to inflammatory cell infiltration (26). Primary lung cancer consistently evolves from pre-invasive lesions (AIS/AAH) that manifested as pure GGO (27) at the early stage. A *post-hoc* analysis (24) found that a solid consistency and increasing size were statistically associated with malignancy.

In addition, our study found that metastatic nodules tended to be round or oval, consistent with previous research (28). We speculate that as metastatic nodules often exhibit a largely uniform growth rate and homogenous invasion in all directions, these features contribute to a round or quasi-circular contour, whereas non-metastatic lesions, including benign lesions and primary lung cancer, have irregular shapes due to uneven growth rates at various sites (26). Thus, short-interval CT follow*-*up is highly recommended for IPNs larger than 5 mm in diameter with solid components and approximately regular margins detected on preoperative chest CT.

In addition to the identification of morphological features, the use of texture analysis is a strength of our study. Previous studies have demonstrated that texture analysis can not only distinguish malignant nodules from benign ones (18) but also differentiate *in situ* and minimally invasive lung adenocarcinoma subtypes (19). These studies have shown that texture parameters can reveal the underlying histological changes in tissue below the resolution of the given modality or protocol. In this study, we found that the W-average ratio, W-mean, and W-median, which represent the zone of CT attenuation within the ROI, were substantially higher in the metastasis group than in the non-metastasis group. Thus, short-interval CT follow*-*up is highly recommended for IPNs larger than 5 mm in diameter with solid components and approximately regular margins detected on preoperative chest CT. This speculation is also in line with another finding of our study that vascular convergence was more common and the enhancement degree was higher in the metastasis group than in the non-metastasis group. However, as texture analysis is a mathematical method, the biological mechanisms underlying the textural features are complex and not completely understood (29). In cases where vascular convergence or the enhancement degree is insufficient to differentiate metastatic lesions, the values from the CT attenuation zone might exhibit local variation and more sensitive preservation of spatial information (30).

Another finding of our study was that the whole-lesion texture analysis outperformed the single-slice analysis in evaluating pulmonary nodules, consistent with a previous study (31). Whole-lesion analysis may provide a more comprehensive understanding of the stereo structure of the whole lesion and thereby reflect the integral heterogeneity better than can single-slice analysis. Despite the time-consuming process of the contouring around the whole lesion, it seems more cost-efficient to use this method as it provides improved prediction relative to single-slice analysis and a more definite diagnosis, allowing timely treatment and maximizing the benefits to the patient.

For clinical use, we constructed a risk stratification nomogram for the clinician to predict the risk of PM for an individual CRC patient. As the early and accurate diagnosis of pulmonary metastasis has been recognized as one of the most important steps in treating potential curable lesions with surgery, we propose that patients with a high risk of PM be considered candidates for thoracotomy for resectable lesions to enhance local control and improve the survival rate. We also hope this model can help low*-*risk patients avoid aggressive follow*-*up and reduce the burden of radiation exposure. We believe that the clinical use of the nomogram can contribute to reliable diagnoses and help clinicians optimize therapeutic plans for IPNs at an early stage after detection.

Our study has several limitations. First, as a retrospective study, thin-slice contrast-enhanced CT images from our database were used, which limited the number of cases for analysis. And the inclusion and exclusion criteria also limits the implementation of the study in clinical practice. Second, only histogram parameters were extracted in this study. In our previous research (20), 203 radiomic features, including first- and second-order parameters, attained a prognostic value in the differentiation of pulmonary metastasis with an AUC of 0.888, which is slightly higher than that obtained using the histogram parameters (AUC = 0.887). However, the process of extracting radiomic features through MATLAB is intricate and demanding for radiologists and clinicians, which constrains its clinical utilization. The volume histogram analysis performed here allowed the simple, efficient, and automatic acquisition of a density histogram and achieved an accuracy comparable to that of the radiomics analysis. Thus, volume histogram analysis may be more appropriate for imperative clinical decisions, and radiomics analysis can be used as a supplementary method when needed. Another limitation is that the development and validation were performed in a single institution. External validation and multi-center clinical trials are therefore needed for further generalization.

In conclusion, the results of our study demonstrated that histogram parameters may serve as non-invasive imaging biomarkers for differentiating pulmonary metastasis from non-metastatic lesions. When complemented with morphological features, the morphological-histogram nomogram can greatly benefit the diagnosis of pulmonary metastasis in CRC patients.

## Data Availability Statement

The datasets generated for this study are available on request to the corresponding author.

## Ethics Statement

The studies involving human participants were reviewed and approved by Medical ethics committee of Fudan University Shanghai Cancer Center. Written informed consent for participation was not required for this study in accordance with the national legislation and the institutional requirements.

## Author Contributions

TT and SW carried out the concepts and design of the study. DS provided the patients information. YL confirmed the pathology results. XE, YY, HL, and JW provided assistance for data acquisition and statistical analysis. WP provided the permission of imaging acquisition. SW and TH carried our literature research and manuscript editing. TH and SW contributed equally to this work. All authors have reviewed the final version of the manuscript and approved it for publication.

### Conflict of Interest

The authors declare that the research was conducted in the absence of any commercial or financial relationships that could be construed as a potential conflict of interest. The reviewer DY declared a shared affiliation, though no other collaboration, with the authors to the handling Editor.
