# Supplementary material for: CT Morphological Features Integrated With Whole-Lesion Histogram Parameters to Predict Lung Metastasis for Colorectal Cancer Patients With Pulmonary Nodules
Source: Front Oncol. 2019 Nov 19;9:1241. doi: 10.3389/fonc.2019.01241 (PMC6877751; doi:10.3389/fonc.2019.01241)
Supplement: Supplementary file 1 [file Presentation_1.pdf]

Supplementary Material

Figure 1

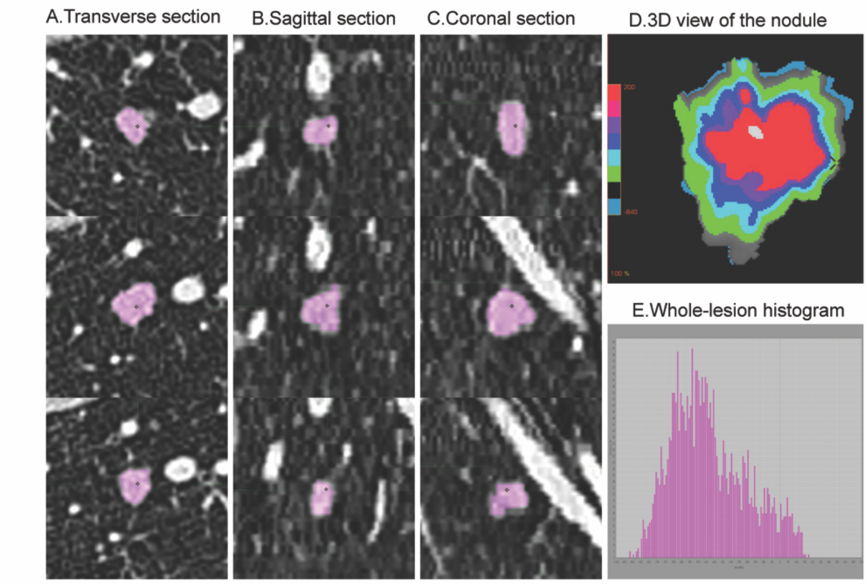

An example of ROI delineation.

Table S1 Definition of CT morphological features

Characteristics	Definition and scoring
CLINICAL FEATURES	
**Age**	Patients' age when the pulmonary nodule was firstly detected by CT examination
**Gender**	Patients' gender
**1**	Male
**2**	Female
**Primary tumor**	Location of colorectal cancer
**1**	Left colon
**2**	Right colon
**3**	Rectum
LOCATION	
**Distribution**	Central location(>2cm away from the pleural) or peripheral location(≤2cm away from the pleural)
**1**	Central location
**2**	peripheral location
**Lobe location**	Lobe location of the tumor
**1**	Right upper lobe
**2**	Right middle lobe
**3**	Right lower lobe

**4**	Left upper lobe
**5**	Left lower lobe
**Nodules number**	Nodules with the longest diameter ≥5mm were counted
**1**	One nodule
**2**	Double nodules
**3**	Multiple nodules
SIZE CATEGORY	
**Size**	The largest length of the target lesion
**1**	5-10mm
**2**	11-15mm
**3**	16-20mm
**Long-axis diameter**	Longest diameter of the tumor
**Short-axis diameter**	Longest perpendicular diameter in the same section
ATTENUATION	
**Density**	Solid nodule or ground-glass opacity(GGO)
**1**	Pure GGO
**2**	Mixed GGO
**3**	Solid nodule
**Calcification**	Any patterns of calcification in the tumor
**0**	No
**1**	Yes
SHAPE	
**Contour**	The overall shape of roundness
**1**	Round-like contour
**2**	Oval-like contour
**3**	Somewhat irregular
**4**	Irregular
**Lobulation**	A portion of a lesion's surface that showed a wavy or scalloped configuration
**0**	None
**1**	Lobulation number less than 3
**2**	Lobulation number between 4 and 6
**3**	Lobulation number more than 6
**Border**	Well- or ill-defined border
**1**	Well defined border
**2**	Everything else between 1 and 3
**3**	Poorly defined border
**Spiculation**	Lines radiating from the margins of the tumor
**0**	None
**1**	Fine spiculation
**2**	Coarse spiculation

INTERNAL STRUCTURE	
**Air bronchogram**	Tubelike or branched air structure within the tumor
**0**	No
**1**	Yes
**Concavity**	Concave cuts
**0**	None
**1**	Slight concavity
**2**	Deep concavity
**Vocule sign**	The presence of air in the tumor
**0**	No
**1**	Yes
ADJACNET FINDINGS	
**Fissure attachment**	Nodule attaches to the fissure
**0**	No
**1**	Yes
**Pleural attachment**	Nodule attaches to the pleura other than fissure
**0**	No
**1**	Yes
**Pleural retraction**	Retraction of the pleura toward the nodule
**0**	No
**1**	Yes
ASSOCIATED FEATURES	
**Vascular convergence**	Convergence of vessels to the tumor
**0**	No
**1**	Yes
**Lymphadenopathy**	Thoracic lymph nodes (hilar or mediastinal) with short-axis diameter greater than 1 cm
**0**	No
**1**	Yes
**Enhancement degree**	Degree of enhancement on contrast-enhanced CT images(lesion CT value/Aorta CT value)

Table S2 Comparison between primary cohort and validation cohort

Characteristics	Primary cohort	Validation cohort	p
**Metastasis**			0.365
**0**	73(53.7%)	28(46.7%)	
**1**	63(46.3%)	32(53.3%)	
**Age**	59.06±10.912	57.22±10.612	0.273
**Gender**			0.741
**1**	76(55.9%)	32(53.3%)	
**2**	60(44.1%)	28(46.7%)	
**Primary tumor**			0.685
**1**	32(23.5%)	15(25.0%)	
**2**	25(18.4%)	8(13.3%)	
**3**	79(58.1%)	37(61.7%)	
**Distribution**			0.805
**1**	102(75.0%)	44(73.3%)	
**2**	34(25.0%)	16(26.7%)	
**Lobe location**			0.263
**1**	31(22.8%)	13(21.7%)	
**2**	22(16.2%)	14(23.3%)	
**3**	32(23.5%)	13(21.7%)	
**4**	17(12.5%)	2(3.3%)	
**5**	34(25.0%)	18(30.0%)	
**Nodules number**			0.689
**1**	57(41.9%)	22(36.7%)	
**2**	33(24.3%)	14(23.3%)	
**3**	46(33.8%)	24(40.0%)	
**Size category**			0.916
**1**	78(57.4%)	34(56.7%)	
**2**	34(25.0%)	14(23.3%)	
**3**	24(17.6%)	12(20.0%)	
**Long-axis diameter**	10.690±4.242	10.607±4.454	0.901
**Short-axis diameter**	7.579±6.736	7.017±2.903	0.536
**Density**			0.948
**1**	12(8.8%)	6(10.0%)	
**2**	17(12.5%)	8(13.3%)	
**3**	107(78.7%)	46(76.7%)	
**Calcification**			0.602
**0**	132(97.1%)	59(98.3%)	
**1**	4(2.9%)	1(1.7%)	
**Contour**			0.066
**1**	12(8.8%)	2(3.3%)	

**2**	41(30.1%)	10(16.7%)	
**3**	48(35.3%)	30(50.0%)	
**4**	35(25.7%)	18(30.0%)	
**Lobulation**			0.761
**0**	66(48.5%)	25(41.7%)	
**1**	35(25.7%)	16(26.7%)	
**2**	20(14.7%)	12(20.0%)	
**3**	15(11.0%)	7(11.7%)	
**Border**			0.954
**1**	30(22.1%)	14(23.3%)	
**2**	67(49.3%)	30(50.0%)	
**3**	39(28.7%)	16(26.7%)	
**Spiculation**			0.276
**0**	76(55.9%)	39(65.0%)	
**1**	58(42.6%)	19(31.7%)	
**2**	2(1.5%)	2(3.3%)	
**Air bronchogram**			0.576
**0**	129(94.9%)	58(96.7%)	
**1**	7(5.1%)	2(3.3%)	
**Concavity**			0.739
**0**	105(77.2%)	49(81.7%)	
**1**	16(11.8%)	5(8.3%)	
**2**	15(11.0%)	6(10.0%)	
**Vocule sign**			0.663
**0**	105(77.2%)	48(80.0%)	
**1**	31(22.8%)	12(20.0%)	
**Fissure attachment**			0.793
**0**	124(91.2%)	54(90.0%)	
**1**	12(8.8%)	6(10.0%)	
**Pleural attachment**			0.023*
**0**	84(61.8%)	47(78.3%)	
**1**	52(38.2%)	13(21.7%)	
**Pleural retraction**			0.564
**0**	111(81.6%)	51(85.0%)	
**1**	25(18.4%)	9(15.0%)	
**Vascular convergence**			0.884
**0**	71(52.2%)	32(53.3%)	
**1**	65(47.8%)	28(46.7%)	
**Lymphadenopathy**			0.67
**0**	129(94.9%)	56(93.3%)	
**1**	7(5.1%)	4(6.7%)	

**Enhancement degree**	0.043±0.463	0.151±1.127	0.344
**S-ASD**	-2.942±12.810	-0.818±8.057	0.238
**S-STD**	149.474±85.925	130.025±63.189	0.117
**S-Average ratio**	0.853±1.174	0.774±0.911	0.645
**S-Mean**	-138.862±335.183	-54.635±129.725	0.061
**S-Skewness**	-0.348±1.077	-0.357±1.148	0.96
**S-Kurtosis**	1.068±4.083	1.558±4.781	0.465
**S-Area**	0.988±0.448	0.973±0.426	0.818
**S-Volume**	0.853±1.174	0.774±0.911	0.645
**S-Median**	-168.560±284.811	-155.480±256.045	0.76
**S-Maximum**	174.650±350.718	162.680±416.102	0.836
**S-Minimum**	-617.740±246.002	-574.920±257.463	0.27
**W-ASD**	296.735±678.869	207.384±249.451	0.324
**W-STD**	149.620±85.682	129.695±63.118	0.107
**W-Average ratio**	1.261±0.589	1.240±0.501	0.813
**W-Mean**	-174.432±263.197	-166.792±242.295	0.848
**W-Skewness**	-0.360±1.035	-0.344±1.157	0.923
**W-Kurtosis**	3.937±3.912	4.554±4.878	0.347
**W-Area**	142.091±193.520	133.287±161.378	0.758
**W-Volume**	255.038±445.507	218.210±316.000	0.563
**W-Median**	-163.971±278.803	-155.583±255.981	0.843
**W-Maximum**	173.680±350.142	169.200±405.687	0.938
**W-Minimum**	-590.240±257.365	-556.000±278.079	0.403

*$p<0.05$

Table S3 Comparison of morphological features of lung metastasis (PM) and non- metastasis (NM) in the training and validation datasets

	Training dataset			Validation dataset		
Characteristics	NM	PM	p	NM	PM	p
Age	59.82±10.530	58.17±11.360	0.382	58.54±12.188	56.06±9.055	0.372
Gender			0.676			0.316
1	42(57.5%)	34(54.0%)		13(46.4%)	19(59.4%)	
2	31(42.5%)	29(46.0%)		15(53.6%)	13(40.6%)	
Primary tumor			0.082			0.198
1	21(28.8%)	11(17.5%)		10(35.7%)	5(15.6%)	
2	16(21.9%)	9(14.3%)		3(10.7%)	5(15.6%)	
3	36(49.3%)	43(68.3%)		15(53.6%)	22(68.8%)	
Distribution			0.091			0.042*
1	59(80.8%)	43(68.3%)		24(85.7%)	20(62.5%)	
2	14(19.2%)	20(31.7%)		4(14.3%)	12(37.5%)	
Lobe location			0.025*			0.507
1	12(16.4%)	19(30.2%)		7(25.0%)	6(18.8%)	
2	12(16.4%)	10(15.9%)		4(14.3%)	10(31.3%)	
3	23(31.5%)	9(14.3%)		8(28.6%)	5(15.6%)	
4	12(16.4%)	5(7.9%)		1(3.6%)	1(3.1%)	
5	14(19.2%)	20(31.7%)		8(28.6%)	10(31.3%)	
Nodules number			0.886			0.169
1	32(43.8%)	25(39.7%)		11(39.3%)	11(34.4%)	
2	17(23.3%)	16(25.4%)		9(32.1%)	5(15.6%)	

3	24(32.9%)	22(34.9%)		8(28.6%)	16(50.0%)	
Size category			<0.001*	(0.0%)	(0.0%)	0.095
1	56(76.7%)	22(34.9%)		20(71.4%)	14(43.8%)	
2	10(13.7%)	24(38.1%)		4(14.3%)	10(31.3%)	
3	7(9.6%)	17(27.0%)		4(14.3%)	8(25.0%)	
Long-axis diameter	8.988±3.493	12.662±4.200	<0.001*	9.221±4.422	11.819±4.180	0.023*
Short-axis diameter	5.825±2.144	9.611±9.253	0.001*	5.682±2.031	8.184±3.071	<0.001*
Density			<0.001*			0.002*
1	12(16.4%)	0(0.0%)		6(21.4%)	0(0.0%)	
2	15(20.5%)	2(3.2%)		6(21.4%)	2(6.3%)	
3	46(63.0%)	61(96.8%)		16(57.1%)	30(93.8%)	
Calcification			0.059			0.281
0	69(94.5%)	63(100.0%)		27(96.4%)	32(100.0%)	
1	4(5.5%)	0(0.0%)		1(3.6%)	0(0.0%)	
Contour			0043*			0.011*
1	2(2.7%)	10(15.9%)		1(3.6%)	1(3.1%)	
2	21(28.8%)	20(31.7%)		2(7.1%)	8(25.0%)	
3	29(39.7%)	19(30.2%)		11(39.3%)	19(59.4%)	
4	21(28.8%)	14(22.2%)		14(50.0%)	4(12.5%)	
Lobulation			0.267			0.179
0	40(54.8%)	26(41.3%)		15(53.6%)	10(31.3%)	
1	18(24.7%)	17(27.0%)		6(21.4%)	10(31.3%)	
2	10(13.7%)	10(15.9%)		3(10.7%)	9(28.1%)	
3	5(6.8%)	10(15.9%)		4(14.3%)	3(9.4%)	
Border			<0.001*			<0.001*

1	28(38.4%)	2(3.2%)		13(46.4%)	1(3.1%)	
2	24(32.9%)	43(68.3%)		10(35.7%)	20(62.5%)	
3	21(28.8%)	18(28.6%)		5(17.9%)	11(34.4%)	
Spiculation			0.217			0.363
0	39(53.4%)	37(58.7%)		18(64.3%)	21(65.6%)	
1	34(46.6%)	24(38.1%)		10(35.7%)	9(28.1%)	
2	0(0.0%)	2(3.2%)		0(0.0%)	2(6.3%)	
Air bronchogram			0.032*			0.178
0	72(98.6%)	57(90.5%)		28(100.0%)	30(93.8%)	
1	1(1.4%)	6(9.5%)		0(0.0%)	2(6.3%)	
Concavity			0.127			0.268
1	55(75.3%)	50(79.4%)		24(85.7%)	25(78.1%)	
2	12(16.4%)	4(6.3%)		3(10.7%)	2(6.3%)	
3	6(8.2%)	9(14.3%)		1(3.6%)	5(15.6%)	
Vocule sign			0.136			0.698
0	60(82.2%)	45(71.4%)		23(82.1%)	25(78.1%)	
1	13(17.8%)	18(28.6%)		5(17.9%)	7(21.9%)	
Fissure attachment			0.121			0.49
0	64(87.7%)	60(95.2%)		26(92.9%)	28(87.5%)	
1	9(12.3%)	3(4.8%)		2(7.1%)	4(12.5%)	
Pleural attachment			0.596			0.967
0	47(64.4%)	37(58.7%)		22(78.6%)	25(78.1%)	
1	26(35.6%)	26(41.3%)		6(21.4%)	7(21.9%)	
Pleural retraction			0.252			0.885
0	57(78.1%)	54(85.7%)		24(85.7%)	27(84.4%)	

1	16(21.9%)	9(14.3%)		4(14.3%)	5(15.6%)	
Vascular convergence			0.092			0.284
0	43(58.9%)	28(44.4%)		17(60.7%)	15(46.9%)	
1	30(41.1%)	35(55.6%)		11(39.3%)	17(53.1%)	
Lymphadenopathy			0.032*			0.369
0	72(98.6%)	57(90.5%)		27(96.4%)	29(90.6%)	
1	1(1.4%)	6(9.5%)		1(3.6%)	3(9.4%)	
Enhancement degree	0.011±0.036	0.271±0.632	0.459	0.035±0.116	0.284±1.652	0.398

Note: Chi-square tests were used to compare the differences in categorical variables while a two-sample t-test was used to compare the differences in continuous variables.

Abbreviations: NM, non-metastasis group; PM, lung metastasis group.

Table S4 Inter-observer variability of CT morphological features

Characteristics	ICC	95%CI
**Distribution**	1	1.000-1.000
**Lobe location**	1	1.000-1.000
**Nodules number**	1	1.000-1.000
**Size category**	0.996	0.994-0.997
**Long-axis diameter**	0.961	0.948-0.970
**Short-axis diameter**	0.936	0.916-0.952
**Density**	0.883	0.847-0.910
**Calcification**	1	1.000-1.000
**Contour**	0.834	0.785-0.872
**Lobulation**	0.907	0.879-0.929
**Border**	0.779	0.717-0.829
**Spiculation**	0.758	0.692-0.812
**Air bronchogram**	0.896	0.864-0.920
**Concavity**	0.957	0.953-0.973
**Vocule sign**	0.741	0.670-0.798
**Fissure attachment**	0.843	0.797-0.897
**Pleural attachment**	0.943	0.925-0.956
**Pleural retraction**	0.803	0.746-0.803
**Vascular convergence**	0.89	0.856-0.916
**Lymphadenopathy**	0.95	0.934-0.962
**Enhancement degree**	0.747	0.677-0.893
